# Supplementary material for: Early evidence of molariform hypsodonty in a Triassic stem-mammal
Source: Nat Commun. 2019 Jun 28;10:2841. doi: 10.1038/s41467-019-10719-7 (PMC6598982; doi:10.1038/s41467-019-10719-7)
Supplement: Supplementary file 1 — Supplementary Information [file 41467_2019_10719_MOESM1_ESM.pdf]

Supplementary Information

Early evidence of molariform hypsodonty in a Triassic stem-mammal

Melo et al.

Supplementary Note 1: Institutional abbreviations

MCN-PV, Museu de Ciências Naturais, Fundação Zoobotânica do Rio Grande do Sul, Porto Alegre, Brazil; **UA**, Université d'Antananarivo, Antananarivo, Madagascar; **UFRGS-PV-T**, Universidade Federal do Rio Grande do Sul, Porto Alegre, Brazil.

Supplementary Note 2: Brief anatomical description

MCN-PV-10216 (Supplementary Fig. 2c)

MCN-PV-10216 is a small-sized lower jaw (estimated 90 mm anteroposterior length; 67 mm from symphysis to base of angular process), found with a vertebra and rib fragments attached to the right dentary. The mandibular rami were pressed together and slightly twisted close to the symphysis. Both rami are also fractured posterior to the postcanine series, causing the left coronoid process to be missing. On the left side, the angle of the dentary is also rotated and crushed against the tooth-row, deforming the posterior-most postcanines. The symphysis is fused and extends posteriorly to the level of the second postcanine. The angular process is strongly projected posteriorly, dorsal to it, is a wide emargination on the posterior border of the dentary leading to the coronoid process. The coronoid process does not hide the last postcanine (pc6) in labial view, although there is a possible alveolus posterior to pc6, indicative of an additional erupting postcanine. The lower dental formula is i3, c1, pc6 + ?1.

The incisors are procumbent, although most of the crowns are broken at the base and missing. In cross-section, i1 can be characterized as salinon-shaped[1], but the others are more difficult to assess. The canine is small (estimated crown height of less than 9 mm) and procumbent.

The postcanines are curved and canted distally, with roughly rectangular shape in cross-section, long axis oriented mesio-distally, and a longitudinal sulcus along the middle of the lingual side. Posteriorly in the tooth-row, the teeth are progressively larger and less worn. The anterior-most postcanine (pc1) is broken at the base in both mandibles and differs from the other postcanines by having a round cross-section (approx. 1 mm diameter). Both pc2 and pc3 are more worn on the labial side, resulting in low labial ridges and relatively high lingual cusps of the (mesially positioned) transverse ridge. The central occlusal basin is shallow. The distal heel is very pronounced in all postcanines, contacting the mesial face of the adjacent postcanine almost to the apex of the transverse ridge. The degree of wear is smaller in pc4 and pc5, accordingly, the labial ridge is higher, the labial and lingual cusps of the transverse ridge are subequal in height, and the occlusal basin is deeper. The enamel is not distinguishable in pc1-4, but it can be seen as a thin translucent layer around the crowns of pc5 and pc6. Differing from

the generally flat external surfaces of the other postcanines, pc6 has a more convex shape of the outer enamel cover, which gradually thins and transitions to the rugose aspect of cementum. The maximum height of the enamel band in pc6 is 2.5 mm, at the mesio-lingual cusp and at the labial side of distal heel.

UFRGS-PV-1165-T (Fig. 1i)

Includes the right side of a snout, found in association with an edentulous lower jaw (Melo *et al.*[2], Fig. 6A) and cranial fragments of more than one individual. The snout is comparable in size and morphology to the complete skull UFRGS-PV- 1164-T (Melo *et al.*[2], Figs. 2-5), both having 87 mm maxillary length (between the posterior contact with the transverse process of the pterygoid to the anterior contact to the premaxilla). The lateral face of the maxilla was removed to expose the intra-alveolar portion of the postcanines.

Each of the five postcanines is curved mesially. The teeth are open-rooted and tubular in appearance, with no cervix separating crown and root. The root apex has the same anteroposterior width as the crown. Although the postcanines vary in size and the anterior teeth (pc1-2) are more worn, (as in all specimens of *Menadon*), the intra-alveolar height is approximately constant. Enamel is not present.

Around each postcanine, there is periodontal space, filled with minerals, separating the cementum from the alveolar bone. Well-developed interalveolar septa are present between the postcanines.

Supplementary Note 3: Microstructural description

MCN-PV-10221 – *Menadon besairiei* upper postcanine

Ground section - Middle Longitudinal Section (Supplementary Fig. 3b)

The upper postcanine, curved mesially, parallel mesial and distal surfaces, with open pulp cavity. Large portions of the outer mineralized tissue were removed during early mechanical preparation, small areas remain in the distal face.

The section in dominated by orthodentine, with characteristically sigmoid tubules. The dentinal tubules start in the coronal direction from the central portion of the tooth, curving gently towards the outer surface, then coronally approximately halfway, and finally perpendicular again to the CDJ. There is a discontinuity in the dentin in the outer ~500 µm, with a lighter band (~150 µm) separating the external and internal layers, in the outer layer, the dentinal tubules are more diffuse and arranged almost perpendicularly to the CDJ. In the periphery of the dentin, there is a brighter, thin (30 µm) layer, where the tubules branch out before fading.

External to the hyaline dentin, a layer of alternating cellular and acellular cementum is approx. 230 µm in the deepest preserved patch, thinning to approx. 100 µm at the level of the pulp chamber. In the coronal region, there are ill-defined incremental lines in the cementum and Sharpey fibres light can be seen in the external layers under polarized.

Dentine structural lines can be seen along the section, perpendicular to the dentinal tubules, markedly in the medial and root apical portion of the tooth, and more faintly in the external layer of dentin. Lighter broad bands coincide with the primary curvature of the dentinal tubules, corresponding to Schreger lines. Contour lines are present in most of the tooth, and incremental von Ebner's lines and longer period Andresen lines can be seen more clearly in the internal dentine, sometimes coincident with the contour lines. The daily lines of von Ebner are 5-7 µm wide, and about 10 are disposed between two Andresen lines.

Scanning electron microscope - Middle Longitudinal Section

The features of orthodentine are clearly recognizable in the SEM micrographs. The internal layer of dentine has a coarser texture compared to the external, becoming smoother abruptly, despite the gradual change in the orientation of the tubules (Fig. 2B). The tubules in the external dentine are more ramified and also seem to be slightly thinner than in the internal dentine. This may indicate a sudden change in the proportion of types of dentine, the inner portion being composed chiefly of intertubular dentine, the outer richer in peritubular dentine.

In all preserved parts of the cementum, there are numerous cementocytes lacunae, but rest lines or internal layers are not clearly defined. The cementum matrix is fibrous in appearance, with small intrinsic fibres arranged disorganizedly and larger extrinsic (Sharpey's) fibres arranged perpendicularly or obliquely to the surface[3]. The Sharpey's fibres are well mineralized, very few lacking a mineralized core, and, although more common in the periphery, can be seen penetrating to the interior of the cementum, in between the cementocytes, usually in an oblique apical direction.

MCN-PV-10343 – *Menadon besairiei* lower postcanine

Ground section - Middle Longitudinal Section (Supplementary Fig. 3f)

The lower postcanine is curved distally, the mesial and distal surfaces are parallel, with open pulp cavity. Part of the mesial side of the tooth did not resist the lamination process, the cementum and exterior dentine are better-preserved in the mesial root apex. The microstructure is also visible on the distal side of the tooth, but it is severely altered by diagenesis.

Orthodentine occupies most of the section, divided in larger internal and narrower external portions, as in the upper postcanines. The external dentine is approx. 250 µm wide on the distal side and 500 µm on the mesial, thinning in the root apical portion to 300 µm. Large areas of the external dentine were altered in the distal side, making the tubules not visible (also perceptible macroscopically as translucent zones). Curiously, undulating lines of von Ebner are visible in these areas (2.5- 6 µm). On the whole distal side, along the boundary from the internal to the external dentine, there seems to be a zone of interglobular dentine.

The cementum on the distal side is mostly obscured by diagenetic alteration in the form of coarse dark fibres, sub-parallel to the surface, in certain points penetrating into the dentine (more than 100 µm). Portions of the underlying cellular cementum can be seen in more apical areas. The average thickness of the cementum in the distal side is 60 µm, and 180 µm in preserved part of the mesial side.

The mesial apical extremity of the tooth is formed by orthodentine comparable to the external dentine of more coronal parts, with von Ebner's lines forming a wavy pattern, and a thin (100 µm) layer of cellular cementum. In the outer side, striations typical of Sharpey's Fibres are visible under polarized light.

Supplementary Discussion: Tooth Replacement in gomphodont cynodonts (Fig. 3)

From the plesiomorphic, “reptilian”, alternate replacement of non-mammalian synapsids, both probainognathian and gomphodont cynodonts independently developed slower rates of replacement during the Triassic[4–6]. In the probainognathian lineage, this culminated in diphyodonty in mammaliaforms[4], and in continuous sequential replacement in tritylodontids[7–9] and, eventually, in mammals[10].

In the Early and Middle Triassic, gomphodont cynodonts evolved labio-lingually expanded molariform postcanines. These gomphodont-type postcanines often coexisted with sectorial and simpler postcanines, especially in early gomphodonts, such as diademodontids and trirachodontids. Incisor and canine teeth retained the plesiomorphic alternate pattern of replacement, whereas the postcanines developed a more complex tooth succession, which minimized disruptions in the occlusion of gomphodont teeth. Eruption occurred sequentially from front to back of the tooth row, with the posterior sectorials, when present, being replaced by gomphodont or other sectorial teeth[11–13]. Typically, the gomphodont postcanine tooth row shows an anteroposterior increase in size, reflecting ontogenetic growth at the time of eruption.

In the diademodontid *Diademodon*, postcanines of up to six[14] (depending on the interpretation) distinct morphologies were present at the same time, continually replacing each other throughout the animal’s life. Sectorial postcanines erupted at the rear of the tooth row and were replaced sequentially by gomphodont teeth. The anterior-most, consequently oldest, gomphodont teeth were, in turn, replaced by conical postcanines anteriorly, which were not replaced. It remains unclear how the teeth of intermediate morphology (also called subgomphodont) between the sectorial and gomphodont postcanines, fit in this replacement mode[14–17].

The dental replacement of trirachodontids is possibly the most complex, though it has not been studied in such detail as in diademodontids and traversodontids. Most trirachodontids (except *Trirachodon berryi*) have posterior sectorial postcanines during adult life, which can be replaced by gomphodont or other sectorial teeth[18], but juvenile specimens show that an initial generation of cheek teeth was completely sectorial, followed by several waves of replacement. The fast rate of replacement in some small juveniles record the only examples of a gomphodont postcanine in the process of being vertically replaced by another gomphodont postcanine[18,19].

Traversodontids generally lack any sectorial dentition as adults, although some species (*Andescynodon mendozensis, Pascualgnathus polanskii* and *Massetognathus pascuali*) had posterior sectorials, as small juveniles, that were replaced by gomphodont postcanines[20,21]. *Boreogomphodon jeffersoni* is the exception in retaining its sectorial postcanines through

maturity[11]. Because the completely gomphodont series tends to be established from a very young age, few species have large enough ontogenetic samples as to exclude the possibility of early sectorials. In medium to mature specimens, the least worn and latest to erupt postcanine is invariably the most posterior gomphodont tooth, often with one or more posterior postcanines in process of eruption.

In Traversodontidae, as in other gomphodonts, each new gomphodont postcanine added tends to be larger than the previous one. Most species retain many of the older postcanines as they grow (*e.g. Scalenodon*, *Protuberum*, *Massetognathus, Dadadon*, *Andescynodon*, *Pascualgnathus*, *Mandagomphodon* spp.), resulting in an increase in the number of teeth in larger specimens (*Massetognathus, Dadadon*), while others shed the anterior teeth, which can result in fewer postcanines (as in *Exaeretodon argentinus)* or in a more constant number (in *Exaeretodon riograndensis*, *Menadon besairiei*) during ontogeny[2,22–24]. This "treadmill" type of replacement, in which an indefinite number of teeth are added posteriorly, drift anteriorly and are shed at the anterior end of tooth row, was called Continuous Dental Replacement by Gomes Rodrigues *et al.*[10]. To avoid confusion with alternate replacing polyphyodont dentitions, we used the term "Continuous Sequential Replacement".

In adult individuals of many species (*Scalenodon*, *Massetognathus, Dadadon*, *Andescynodon*, *Pascualgnathus*, *Mandagomphodon* spp.), the last postcanine is not the largest in the tooth row, instead, from a certain point, each new postcanine becomes slightly smaller than the previous[13]. This is likely caused by a decrease in the space available to new postcanines, as a result of slower growth rates after reaching reproductive maturity. Histological and morphological evidence show that, contrary to mammaliaforms, most non-mammalian cynodonts had extended ("indeterminate") growth strategies, with overall slower growth in later ontogeny[4,25–30]. In the early mammaliaform *Morganucodon*, the attainment of truncated ("determinate") growth coincided with the evolution of diphyodonty and, possibly, with other mammalian characters[4,29]. *Menadon* differs from all other traversodontids in that the last lower postcanine is exceptionally reduced in relation to the penultimate, marking the stop of horizontal replacement in all but the smallest known mandible (approx. 90 mm; Supplementary Fig. 2c). The reason for such early interruption is unknown, considering that growth continued after the eruption of the last postcanine, as the holotype (UA 10601) is considerably smaller than other specimens (Supplementary Table 3).

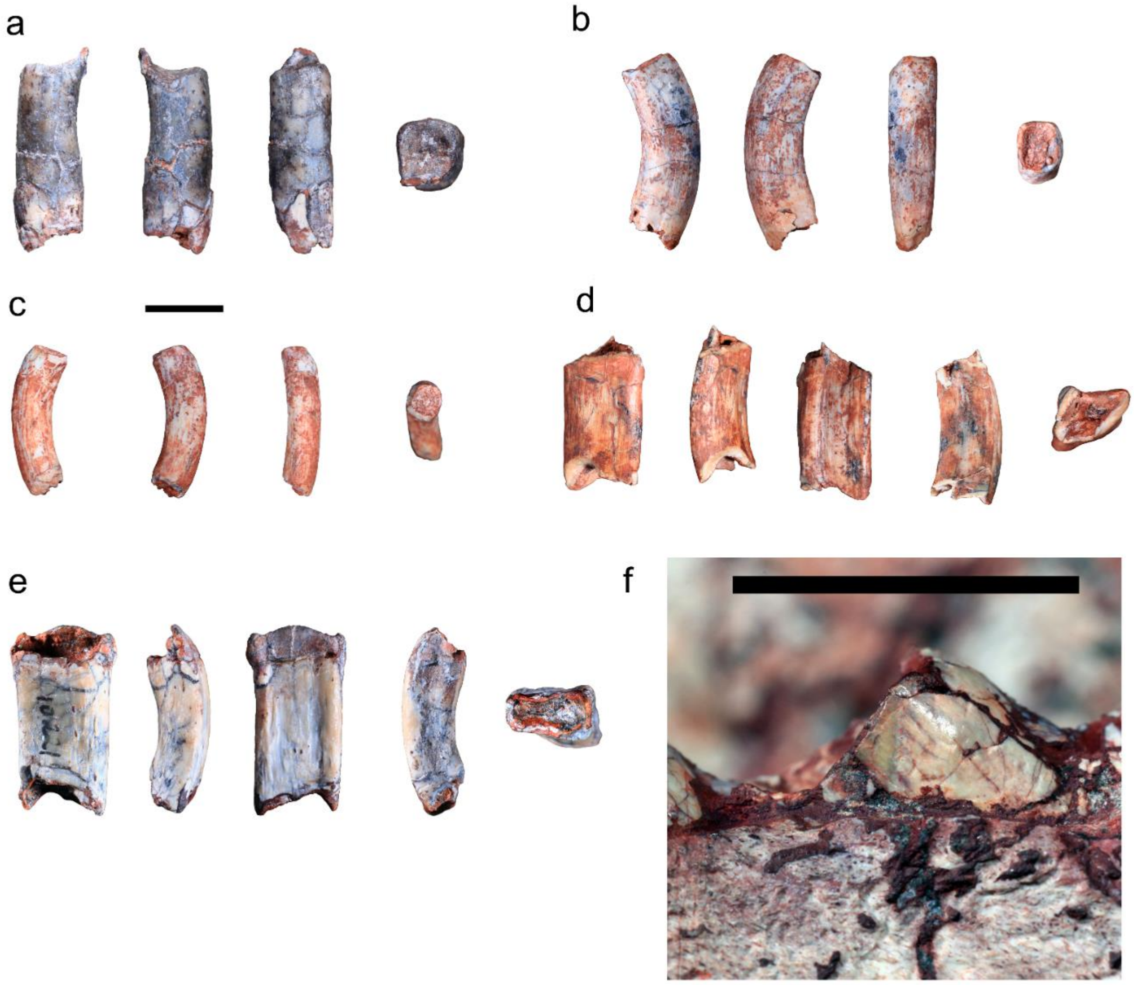

Supplementary Figure 1 | Postcanines of *Menadon besairiei*. a, left lower postcanine in labial, lingual, mesial and occlusal views (MCN-PV-10217 T); **b**, right lower postcanine in labial, lingual, mesial and occlusal views (MCN-PV-10343 T); **c**, left lower postcanine in labial, lingual, mesial and occlusal views(MCN-PV-10339 T); **d**, left upper postcanine in mesial, lingual, distal, labial and occlusal views (UFRGS-PV-1333-T); **e**, right upper postcanine in mesial, lingual, distal, labial and occlusal views (MCN –PV-10221 T); **f**, right lower postcanine (pc4) with residual enamel on the labial side (UFRGS-PV-891-T). Scale bars equal 10 mm, **a-e** are to the same scale.

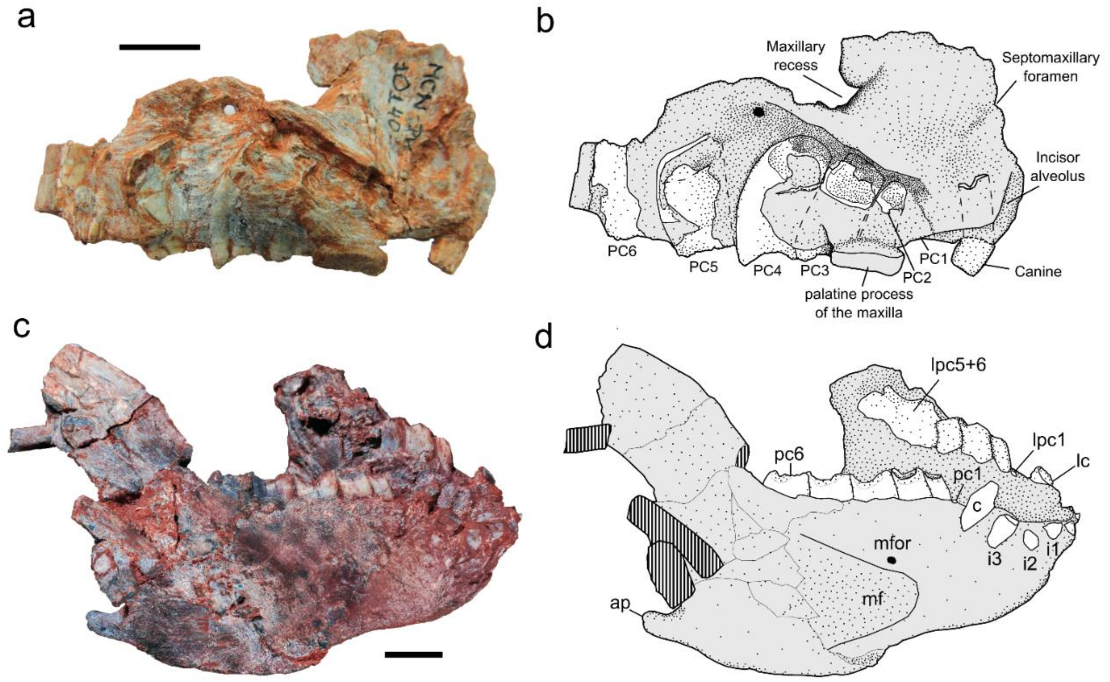

Supplementary Figure 2 | *Menadon besairiei* juvenile individuals. a-b, left maxilla in medial view (MCN-PV-10140 T); **c-d**, paired mandibles in right lateral view (MCN-PV-10216 T). Abbreviations: ap, angular process; c, right canine; i1-3, right incisors; lc, left canine;, lpc1-6, left lower postcanines; mf, masseteric fossa; mfor, mental foramen; pc1-6, right lower postcanines; PC1-6 , upper postcanines. Scale bars equal 10 mm.

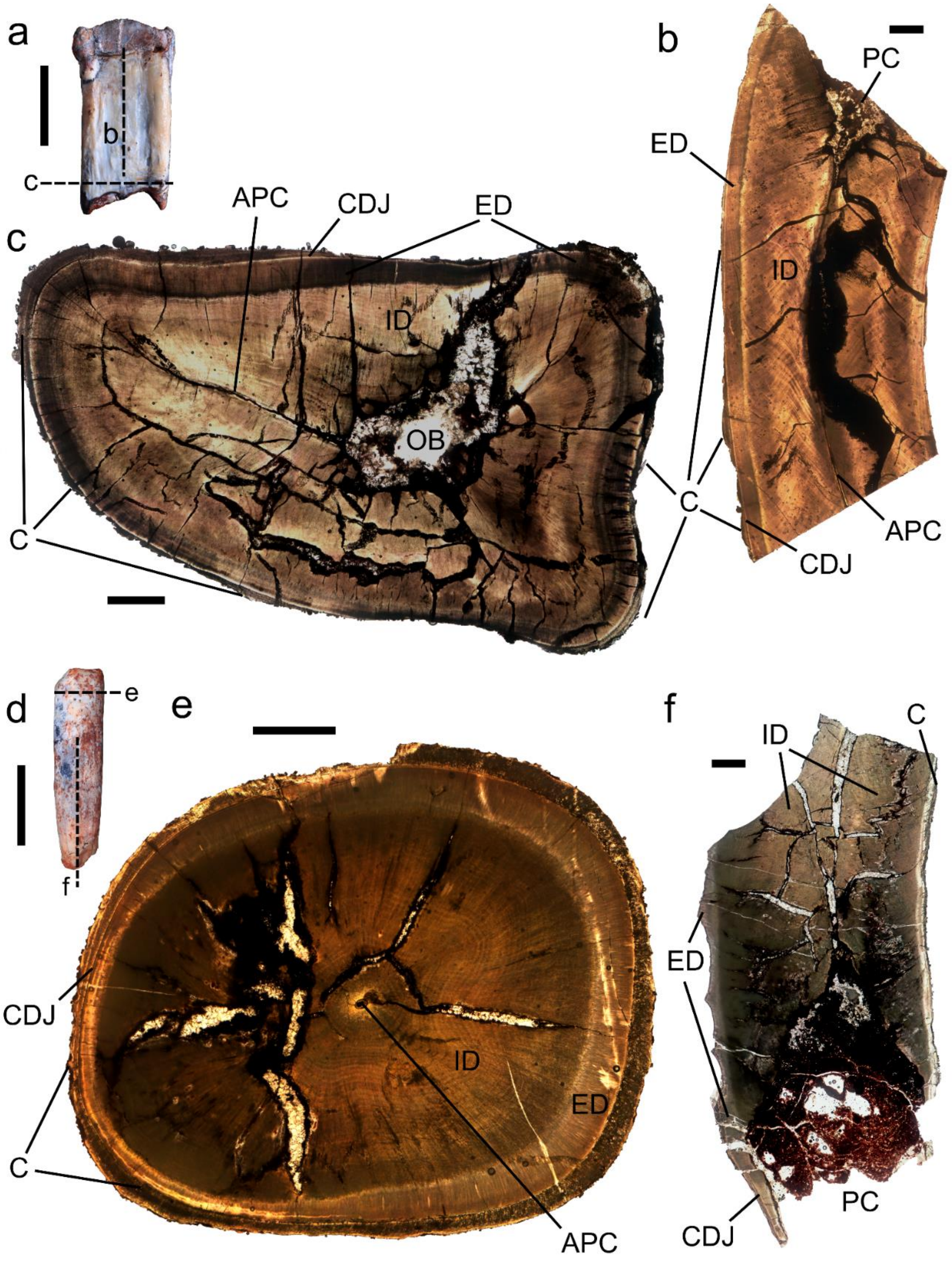

Supplementary Figure 3 | *Menadon besairiei* postcanine tooth microstructure. Upper postcanine (MCN –PV-10221 T). **a,** before sectioning, in distal view, stippled lines indicate position of sections; **b**, longitudinal section; **c**, cross section. Lower postcanine (MCN –PV-10343 T). **d**, before sectioning, in mesial view, stippled lines indicate position of sections; **e**, cross section; **f**, longitudinal section. Abbreviations: APC, atresic pulp chamber; C, cementum; CDJ, cementum-dentine junction; ED, external dentine; ID, internal dentine; OP, occlusal basin; PC, pulp chamber. Scale bars equal 10 mm in a and d and 1mm elsewhere.

Supplementary Table 1. Lower Postcanine Measurements.

Number	Position	Side	AP	AAP	LLW	ALLW	TH
MCN-PV 10343 T	?	R	7	~7.3	6	~5.5	25.4
MCN-PV 10339 T	?	L	5.1	5.1	4.5	4.5	19.8
MCN-PV 10217 T	?	L	8.1	~9	8.3	7.3	~27.3
MCN-PV 10219 T	?	L	6.9	7.2	6.1	6.1	~22.6
MCN-PV 10342 T	?	L	6.1	5.9	4.9	3.5	~22.3
MCN-PV 10216 T	1/1	R	~1	-	~1	-	-
	2/2	R	2.8	-	1.8	-	~17.3
	3/3	R	5.4	-	3.5	-	-
	4/4	R	7	-	4.3	-	~17.20
	5/5	R	7.4	-	4.6	-	-
	6/6	R	7.4	-	5.9	-	~14.3
	1/1	L	~1	-	~1	-	-
	2/2	L	2.6	-	1.7	-	-
	3/3	L	4.4	-	3.4	-	-
	4/4	L	6.7	-	5	-	-
UFRGS-PV-0891-T	1/2	R	4.1	-	3	-	-
	2/3	R	~7	-	5.2	-	-
	3/4	R	~7.5	-	6.6	-	-
	4/5	R	~8	-	~8	-	-
	5/6	R	6.6	-	9.5	-	-
	6/7	R	~5	-	~3	-	-
	3/4	L	~7	-	~7	-	-
	4/5	L	6	-	7.8	-	-
	5/6	L	7.7	-	7.2	-	-
	6/7	L	4.5	-	3.6	-	-
UFRGS-PV-1054-T	1/2	R	2.5	-	2.8	-	-
	2/3	R	6.3	-	4.5	-	-
	3/4	R	~8	-	~7	-	-
	4/5	R	7.6	-	8	-	-
	5/6	R	8.5	-	8.3	-	-
	6/7	R	4.3	-	3.7	-	-
UFRGS-PV-0434-T	1/2	R	~5	-	~3.3	-	-
	2/3	R	~5.4	-	4.7	-	-
	3/4	R	8.1	-	7.2	-	-
	4/5	R	~8.5	-	~7.7	-	-
	6/7	R	~3.3	-	3.8	-	-
	1/2	L	~5.5	-	~~3	-	-
	2/3	L	6	-	5.1	-	-
	3/4	L	8	-	~6	-	-
	4/5	L	~8.5	-	~5.9	-	-
	5/6	L	~10	-	7	-	-
	6/7	L	4.5	-	3.2	-	-

Abbreviations: AAP, root apical mesiodistal length; AP, coronal mesiodistal length; LLW, coronal labiolingual width; ALLW, root apical labiolingual width; TH, tooth height; R, right side; L, left side; ~, deformation; *, estimated; Position, anterior-posterior position in the specimen/ homologous position in the holotype.

Supplementary Table 2. Upper Postcanine Measurements.

Specimen	Position	Side	AP	AAP	LLW	ALLW	TH
MCN-PV 10221 T	?	R	7	7.4	11.3	~13	25
MCN-PV 10218 T	?	L	8	~7.7	11	~11.2	~30
MCN-PV 10340 T	?	R	~7.3	-	~10	-	~13.5
MCN-PV 10220 T	?	R	6.7	~7.5	12.6	~12	~16.3
UFRGS-PV-1333-T	?	L	7	~5.8	9.5	~9	~20.2
MCN-PV 10140 T	1/?2	L	*1.5	-	*1.5	-	?
	2/3	L	~3.5	-	~3.8	-	~10.5
	3/4	L	~4.7	-	~~6	-	~14.5
	4/5	L	~5.5	-	~6.7	-	~17
	5/6	L	~6.5	-	~7.5	-	~15.7
	6/7	L	~10?	-	~3.5	-	-
UFRGS-PV-1164-T	1/4	R	4.5	-	5.3	-	-
	2/5	R	7	-	8.4	-	-
	3/6	R	8	-	10.3	-	-
	4/7	R	8.3	-	11.2	-	-
	5/8	R	6.6	-	12	-	-
	1/4	L	4.9	-	5	-	-
	2/5	L	6.4	-	9.3	-	-
	3/6	L	7.4	-	10	-	-
	4/7	L	8	-	12.6	-	-
	5/8	L	7	-	13	-	-
UFRGS-PV-1165-T	1/4	R	4.4	4.4	4.1	-	18
	2/5	R	5.6	5.5	7	-	20
	3/6	R	7.6	7.3	9	-	22
	4/7	R	7.5	7.6	12.5	-	22
	5/8	R	~7.4	~6.3	12.5	-	24
UFRGS-PV-0865-T	2/5	R	~7.2	-	8	-	-
	3/6	R	~8	-	~7	-	-

Abbreviations: AAP, root apical mesiodistal length of the labial side; AP, coronal mesiodistal length of the labial side; LLW, coronal labiolingual width on the distal side; ALLW, , root apical labiolingual width on the distal side; TH, tooth height; R, right side; L, left side; ~, deformation; *, estimated; Position, position in the specimen/ homologous position in the holotype.

Supplementary Table 3. Cranial and mandibular dimensions.

Specimen	DPL	DTL	BSL	PSL	pc6	PC	pc
MCN-PV 10216 T	67	*90	-	-	No	-	
UFRGS-PV-0891-T	*130	*170	-	-	Yes	-	6
UFRGS-PV-1054-T	125	165	-	-	Yes	-	6
UFRGS-PV-0434-T	150	180	-	-	Yes	-	6
MCN-PV 505 T	~150	~195	-	-	Yes	-	~5-6
UFRGS-PV-0905-T/ 1	*145	*185	-	-	?	-	?
UFRGS-PV-0905-T/ 2	*110	-	-	-	Yes	-	6?
MCN-PV 10140 T	-	-	-	-/57	-	6	-
UFRGS-PV-1164-T	-	-	216	105/86	-	5	-
UFRGS-PV-1165-T/1	-	-	-	*103/87	-	5	-
UFRGS-PV-1165-T/2	130	173	-	-	Yes	-	6
UFRGS-PV-0865-T	-	-	-	/*80	-	5	-
UFRGS-PV-0903-T	-	-	-	/~88	-	5	-
UFRGS-PV-01298-T	-	-	-	*100/~85	-	5	-
UA – 10601 (Holotype)	-	-	158	80.1/?	Yes	8	7
FMNH PR 2444	-	-	-	91.1/?	-	5	-

Abbreviations: DPL, partial dentary length, from the symphysis to base of the angular process; DTL; total dentary length; BSL, basal skull length; PSL, partial skull length (snout/maxilla); pc6, presence of severely reduced sixth lower postcanine; PC, number of upper postcanines or empty postcanine alveoli; pc, number of lower postcanines or empty postcanine alveoli; ~, deformation; *, estimated. All measurements are in millimetres.

Supplementary References

1. Hendrickx, C., Mateus, O. & Araújo, R. A proposed terminology of theropod teeth (Dinosauria, Saurischia). *J. Vertebr. Paleontol.* **35,** e982797 (2015).

2. Melo, T. P., Abdala, F. & Soares, M. B. The Malagasy cynodont *Menadon besairiei* (Cynodontia; Traversodontidae) in the Middle–Upper Triassic of Brazil. *J. Vertebr. Paleontol.* **35,** e1002562 (2015).

3. Boyde, A. & Jones, S. J. Scanning electron microscopy of cementum and Sharpey fibre bone. *Zeitschrift für Zellforsch. und mikroskopische Anat.* **92,** 536–548 (1968).

4. Luo, Z.-X., Kielan-Jaworowska, Z. & Cifelli, R. L. Evolution of dental replacement in mammals. *Bull. Carnegie Museum Nat. Hist.* **36,** 159–175 (2004).

5. Romer, A. S. The Chañares (Argentina) Triassic reptile fauna. VI. A chiniquodontid cynodont with an incipient squamosal-dentary jaw articulation. *Breviora* **344,** 1–18 (1970).

6. Abdala, F., Jasinoski, S. C. & Fernandez, V. Ontogeny of the Early Triassic cynodont *Thrinaxodon liorhinus* (Therapsida): dental morphology and replacement. *J. Vertebr. Paleontol.* **33,** 1408–1431 (2013).

7. Jasinoski, S. C. & Chinsamy, A. Mandibular histology and growth of the nonmammaliaform cynodont Tritylodon. *J. Anat.* **220,** 564–579 (2012).

8. Cui, G. H. & Sun, A. L. Postcanine root-system in tritylodonts. *Vertebr. Palasiat.* **25,** 245 (1987).

9. Gomes Rodrigues, H. & Šumbera, R. Dental peculiarities in the silvery mole-rat: an original model for studying the evolutionary and biological origins of continuous dental generation in mammals. *PeerJ* **3,** e1233 (2015).

10. Gomes Rodrigues, H. *et al.* Continuous dental replacement in a hyper-chisel tooth digging rodent. *Proc. Natl. Acad. Sci.* **108,** 17355–17359 (2011).

11. Liu, J. & Sues, H.-D. Dentition and tooth replacement of *Boreogomphodon* (Cynodontia: Traversodontidae) from the Upper Triassic of North Carolina, USA. *Vertebr. Palasiat.* **48,** 169–184 (2010).

12. Grine, F. E. Postcanine tooth function and jaw movement in the gomphodont cynodont *Diademodon* (Reptilia; Therapsida). *Palaeontol. Africana* **20,** 123–135 (1977).

13. Crompton, A. W. Postcanine occlusion in cynodonts and tritylodonts. *Bull. Br. Museum Nat. Hist. - Geol.* **21,** 29–71 (1972).

14. Osborn, J. W. On Tooth Succession in *Diademodon*. *Evolution (N. Y).* **28,** 141–157 (1974).

15. Ziegler, A. C. A theoretical determination of tooth succession in the therapsid Diademodon. *J. Paleontol.* **43,** 771–778 (1969).

16. Hopson, J. A. in *Early Mammals* (eds. Kermack, D. M. & Kermack, K. A.) 1–21 (Academic Press, 1971).

17. Fourie, S. Tooth replacement in the gomphodont cynodont, *Diademodon*. *S. Afr. J. Sci.* **59,** 211–213 (1963).

18. Sidor, C. A. & Hopson, J. A. Cricodon metabolus (Cynodontia: Gomphodontia) from the Triassic Ntawere Formation of northeastern Zambia: patterns of tooth replacement and a systematic review of the Trirachodontidae. *J. Vertebr. Paleontol.* **37,** 39–64 (2017).

19. Hopson, J. A. A juvenile gomphodont cynodont specimen from the *Cynognathus* Assemblage Zone of South Africa : implications for the origin of gomphodont postcanine morphology. *Palaeontol. Africana* **41,** 53–66 (2005).

20. Goñi, R. & Goin, F. J. Morfologia dentaria y biomecánica masticatoria de los cinodontes (Reptilia, Therapsida) del Triásico argentino: I Andescynodon mendozensis Bonaparte (Traversodontidae). *Ameghiniana* **25,** 139–148 (1988).

21. Martinelli, A. G. On the postcanine dentition of *Pascualgnathus polanskii* Bonaparte (Cynodontia, Traversodontidae) from the Middle Triassic of Argentina. *Geobios* **43,** 629–638 (2010).

22. Abdala, F., Barberena, M. C. & Dornelles, J. A new species of the traversodontid cynodont *Exaeretodon* from the Santa Maria Formation (Middle/Late Triassic) of southern Brazil. *J. Vertebr. Paleontol.* **22,** 313–325 (2002).

23. Goñi, R. & Goin, F. Morfologia dentaria y biomecanica masticatoria de los cinodontes (Reptilia, Therapsida) del Triassico Argentino: II. Exaeretodon frenguellii Cabrera (Traversodontidae). *Ameghiniana* **27,** 327–336 (1990).

24. Abdala, F. & Giannini, N. P. Gomphodont cynodonts of the Chañares Formation: the analysis of an ontogenetic sequence. *J. Vertebr. Paleontol.* **20,** 501–506 (2000).

25. Chinsamy, A. & Abdala, F. Palaeobiological implications of the bone microstructure of South American traversodontids (Therapsida: Cynodontia). *S. Afr. J. Sci.* **104,** 2–7 (2008).

26. O'Meara, R. N., Dirks, W. & Martinelli, A. G. Enamel formation and growth in non-mammalian cynodonts. *R. Soc. open Sci.* **5,** 172293 (2018).

27. Veiga, F. H., Botha-Brink, J. & Soares, M. B. Osteohistology of the non-mammaliaform traversodontids Protuberum cabralense and Exaeretodon riograndensis from southern Brazil. *Hist. Biol.* 1–11 (2018). doi:10.1080/08912963.2018.1441292

28. Botha-Brink, J., Abdala, F. & Chinsamy, A. in *Forerunners of mammals: Radiation, histology, biology* 223–246 (Indiana University Press Bloomington, 2012).

29. O'Meara, R. N. & Asher, R. J. The evolution of growth patterns in mammalian versus nonmammalian cynodonts. *Paleobiology* **42,** 439–464 (2016).

30. Botha-Brink, J., Bento Soares, M. & Martinelli, A. G. Osteohistology of Late Triassic prozostrodontian cynodonts from Brazil. *PeerJ* **6,** e5029 (2018).
